# Current Hospital Topics

**Published:** 1906-06-09

**Authors:** 


### HOSPITAL ADMINISTRATION.

### CONSTRUCTION AND ECONOMICS.

### CURRENT HOSPITAL TOPICS.

#### Munificent Individual Donors.

It is a remarkable and most encouraging feature of voluntary hospital work that each year there is evidence of the great liberality of individuals who take an interest in the welfare of these institutions. When the Duke of Connaught found it impossible to preside at the festival dinner of the Middlesex Hospital, Mr. H. L. Bischoffsheim, whose great generosity during many years is known only to a few, stepped into the breach and sent a contribution of £5,000 to this hospital to repay advances from the bankers, and to meet other immediate expenses, with the view of wiping out any loss due to the postponement of the festival dinner. Knowing as we do how much Mr. Bischoffsheim has done in an infinite variety of ways in the cause of charity during the last twenty years, his gift of £5,000 is indeed munificent, and will, we hope, encourage many others to go and do likewise.

#### Methods of Raising Funds.

Voluntary hospital managers are always in search of new methods of raising funds. We believe, as a matter of experience, that the multiplication of benefit performances, dances, and entertainments of various kinds which has taken place in recent years militates against financial success. Such things are apt to degenerate into a means of gratifying individuals or of promoting their interests rather than those of the great charity for whose benefit they may be nominally held. Boxes again have proved in practice to produce very little money, whilst they give an infinity of trouble and cause no little expense. The new collecting boxes of the London Hospital placed in the stations of the Baker Street and Waterloo Railway, however, merit, if they may not secure, success. They are very attractive in appearance, and the invitation to contribute at least the cost of one second's work of the London Hospital may prove strikingly compelling. We hope this may prove to be the case, and we shall be very glad to publish the results secured at the end of the quarter if the authorities care to supply us with the information.

#### An Endowed Day Scheme.

Brains are not confined to big cities, and great ideas may emanate from the least expected quarters. An earnest worker on the Springfield Mines fields, Nova Scotia, the Rev. W. Charles Wilson, realising the appalling miseries caused to the miners and their families by the want of adequate medical care, a few years ago courageously started the All Saints' Cottage Hospital. With splendid pluck he has laboured continuously to raise the money for buildings and equipment, and has secured, as he deserved, complete success. The truly appalling nature of the difficulties he has had to overcome no doubt made him anxious to provide a permanent income for the maintenance of this miners' hospital. With great ingenuity he evolved an entirely original scheme which he has denominated the Endowed Day Scheme. This plan is based upon the actual cost of a day's maintenance for the whole hospital, which Mr. Wilson has capitalised at 4 per cent. He then asked the heads of families to commemorate some family anniversary, or to perpetuate the memory of some dear member, by contributing a capital sum which, when invested, would yield the amount required to defray the cost of one day's maintenance. This scheme has proved very popular, for in a relatively short time Mr. Wilson has secured the endowment of 304 days, so that he only requires 61 more endowed days to complete the final endowment, and so to assure the permanence of the work irrespective of individual effort. Families and individuals are invited to select their own day, a feature which has added greatly to the popularity of the idea. The scheme has the merit of providing a reserve fund, in the shape of patients' payments and casual contributions, for improving and extending the hospital. In the case of the larger voluntary hospitals the sum required to endow a day would, of course, be considerable, and there it might be advisable to vigorously push a scheme of endowed beds, or to work "the endowed day" scheme by wards, and to organise a committee of ladies for each ward under the direction of the sister. In any case an idea which has proved so fruitful in result in the case of a small community in Nova Scotia may well commend itself to the earnest consideration of all who are responsible for the financial success of a voluntary hospital.

#### Are Hospitals Extravagant?

It is evident from a letter which he has sent to the newspapers that Mr. C. J. Michod, Secretary of the Charing Cross Hospital Emergency Fund, is a man of ingenuity and energy. Setting out to show that hospitals are not extravagant, Mr. Michod takes two branches of expenditure to prove his case—namely, provisions and medical staff. In reference to provisions he states that the price of mutton and beef is 5d. and 6d. per lb., which we must regard as an under-statement. Taking the 23 largest metropolitan general hospitals, the cost of beef at 16 of them exceeded 6d. per lb. In several hospitals the price paid for beef is 7d. per lb., and even higher. He quotes the price of fish at 3½d. per lb., but in eight cases the price paid was 4d. per lb. or upwards; 11d. per lb. is the price he quotes for butter, whereas 14 out of the 23 hospitals paid considerably more than that sum. Dealing with the cost of the medical staff he quotes the case of Charing Cross Hospital, where only one of the eight resident medical officers receives any salary, and then only £100 per annum. This non-payment of resident medical officers is, in our view, a doubtful economy, and we consider that each of them should receive adequate remuneration. Mr. Michod must also be aware that the great increase in the cost per bed at the hospitals in comparatively recent years proves that the items he selects are too limited, for several others have grown out of all proportion to the work done, when the claims of economy are made the first consideration. These omitted items include surgery and dispensary, where in some cases avoidable waste is undoubtedly to be met with at present. Can Mr. Michod guarantee, for instance, that lint is not used in some wards at Charing Cross Hospital for wiping the dishes, and for other purposes than dressing wounds? Domestic expenses, maintenance, salaries and wages, and the cost of the nursing department are other items which show a tendency to continuously increase. All these items demand much closer attention than they apparently receive at present in several hospitals which might be mentioned. In congratulating Mr. Michod on the enterprise he has displayed, and the success which attended the festival dinner presided over by the American Ambassador, we hope he may be able to induce his committee to closely examine the other items of expenditure to which we have called attention. Then, when certain other hospitals follow suit, there is little doubt that the statistical report of the King's Fund will cease to show an excess in the expenditure of several hospitals each year over the average expenditure of the hospitals dealt with in the tables, which amounts to many thousands of pounds.

